# Improving the Connection Between Population-Based and National Clinical Pediatric Cancer Registries—A Pilot Study on Neuroblastoma: The Italian BENCHISTA-Ita Project

**DOI:** 10.3390/cancers17243894

**Published:** 2025-12-05

**Authors:** Fabio Didonè, Andrea Tittarelli, Claudio Tresoldi, Paolo Contiero, Riccardo Capocaccia, Riccardo Haupt, Martina Fragola, Marcella Sessa, Fabio Savoia, Carlotta Sacerdote, Massimo Conte, Gemma Gatta, Laura Botta, BENCHISTA-Ita Working Group

**Affiliations:** 1 Fondazione IRCCS “Istituto Nazionale dei Tumori”, 20133 Milan, Italy; andrea.tittarelli@istitutotumori.mi.it (A.T.);; 2 Editorial Board, Epidemiologia & Prevenzione Journal, Inferenze Scarl, Via Ricciarelli 29, 20148 Milan, Italy; capocaccia.riccardo@gmail.com; 3 IRCCS Istituto Giannina Gaslini, 16147 Genoa, Italy; riccardohaupt@gaslini.org (R.H.);; 4 Biostatistics Unit, Scientific Directorate, IRCCS Istituto Giannina Gaslini, 16147 Genoa, Italy; martinafragola@gaslini.org; 5 Campania Childhood Cancer Registry, 80129 Naples, Italy; m.sessa@santobonopausilipon.it (M.S.);; 6 Azienda Ospedaliera Citta’ della Salute e della Scienza di Torino, 10126 Turin, Italy; carlotta.sacerdote@cpo.it

**Keywords:** neuroblastoma, data linkage, clinical registries, stage at diagnosis, population-based cancer registries

## Abstract

**Background**: Childhood cancers (CC) are rare and treatable; however, survival rates vary across Europe, with poorer outcomes in Eastern countries. Stage at diagnosis is one of the major prognostic factors. The BENCHISTA-Ita project aims to promote the use of the Toronto Guidelines by population-based cancer registries (PBCR) to standardize stage at diagnosis data collection. In Italy, regional cancer registries ensure data quality, while national clinical registries centralize pediatric cancer data. The aim of the study is to promote the linkage between PBCRs and national clinical registries to enhance data completeness. This study presents the results of linking BENCHISTA-Ita neuroblastoma cases with the Italian Neuroblastoma Registry (RINB) as a pilot study. **Methods**: The linkage process involved probabilistic matching using R software, considering variables such as sex, year of birth, and residence at diagnosis. The study included 294 neuroblastoma cases from BENCHISTA-Ita and 578 from RINB, diagnosed between 2013 and 2017. **Results**: Results showed that 272 of 294 BENCHISTA-Ita cases matched with RINB cases, improving the completeness of clinical variables such as stage at diagnosis, N-Myc amplification, and relapse/progression. The linkage increased the completeness of clinical variables in PBCR, such as stage at diagnosis from 81% to 99%, N-Myc from 47% to 86%, and relapse/progression from 68% to 98%. Additionally, the linkage improved RINB’s follow-up completeness from 59% to 97% and added new cases in both PBCR and RINB. **Conclusions**: This linkage demonstrates the potential to enrich both databases, improving data quality and harmonization of cancer indicators. The study highlights the importance of collaboration between PBCRs and clinical registries to ensure comprehensive data collection and enhance more informative population-based studies. Future efforts will focus on expanding the linkage to other national clinical registries.

## Simple Summary

This research aims to improve how childhood cancer data is collected and connected in Italy. By linking two separate databases—one tracking cancer cases across the population and another containing detailed hospital records—the study shows how combining these sources gives a clearer picture of cancer diagnosis, treatment, and outcomes. The pilot focused on neuroblastoma, a common childhood cancer, and successfully matched most cases between the two systems. This helped fill in missing details like cancer stage and relapse information. The goal is to better understand survival differences across regions and ensure all children receive proper care. The findings could lead to more complete national cancer registries and stronger collaboration between hospitals and public health systems, benefiting future research and healthcare planning.

## 1. Introduction

Childhood cancers (CC) are rare, but unlike most rare cancers, they can usually be treated effectively. However, in Europe, large differences between countries for CC survival persist [1,2] and, according to population-based studies, outcomes are usually poor for Eastern European children. The interpretation of these disparities needs to be understood with the standard collection of new clinical variables [3]. In this regard, the Toronto guidelines [4,5,6] aimed to standardize the collection of stage at diagnosis and of other non-stage prognostic clinical variables (NSP) by population-based cancer registries (PBCR).

Gathering information on the incidence and outcomes of childhood cancers is a goal recognized by the WHO [7,8]. In Europe, the registration of childhood cancer at the population level is available nationally in many countries [9], but regrettably, it is completely unavailable in others (e.g., Serbia, Albania, North Macedonia, etc.) [10].

In France, Hungary, Germany, Switzerland, and Romania, despite having partial coverage for all ages or regional registries, there is national coverage for childhood cancers collected by a specialized Pediatric PBCR. In these countries, the connection between pediatric PBCRs and clinical databases, which are rich in clinical information, is well-established, allowing for effective monitoring of clinical trials and studies. By contrast, two other countries, Spain and Italy, have only partial coverage for children.

Nowadays, in Italy, regional PBCRs are presently active, covering areas of different sizes with different periods of starting registration [11]. The data quality is ensured by a specific commission of the Italian Association of Cancer Registries (AIRTUM), and data are periodically published by the International Agency for Research on Cancer (IARC) [12,13]. All Italian regions, except the Abruzzo and Calabria regions, are represented by one or more PBCRs; most are general registries, and two are pediatric registries (covering Campania and Piedmont regions).

On the other hand, national multi-institutional clinical registries centralize data on the diagnosis and treatment of pediatric cancers, even though they are not population-based. These clinical registries are supported by the Italian Association of Pediatric Hematology and Oncology (AIEOP) [14]. The project called ‘National benchmarking of population-based childhood cancer survival by stage at diagnosis’ (BENCHISTA-Ita) is the Italian twin project of the International BENCHISTA project (international benchmarking of childhood cancer survival by tumor stage) [3,15,16]. The two projects share the same goals, specifically, to collect stage at diagnosis for solid pediatric tumors from PBCRs, according to the Toronto Guidelines (TG), and to investigate the different distribution of stage by geographical area and the role of stage in explaining geographical CC survival differences [16,17]. In Italy, outcome variation will be evaluated at the regional level [15].

An additional objective of the BENCHISTA-Ita project is to establish a connection between national hospital registries and PBCRs. This aims to integrate clinical information from PBCRs and improve the completeness of incidence and follow-up data from hospital and population registries [15].

In this paper, we present the results of linking BENCHISTA-Ita neuroblastoma cases, a population-based study, with the Italian Neuroblastoma Registry (RINB) as a pilot study [18,19]. This study assesses the feasibility of the data linkage method, which was performed without any directly identifiable information—such as names or national identification numbers—included in the dataset due to privacy concerns. Moreover, the study assessed the advantages of this linkage on the completeness of the BENCHISTA-Ita project variables and explored the possibility of future use with other national clinical registries.

## 2. Materials and Methods

### 2.1. Sources and Data

#### 2.1.1. BENCHISTA-Ita Database (DB)

BENCHISTA-Ita DB is a population-based core dataset collecting information on 9 solid tumors (osteosarcoma, Ewing sarcoma, Wilms tumor, neuroblastoma, rhabdomyosarcoma, medulloblastoma, ependymoma, astrocytoma, and retinoblastoma) [3,15]. BENCHISTA-Ita data are standardized and comparable across registries and therefore represent the gold standard for the acquisition and the validity of demographic variables, including the follow-up and life status.

**Figure 1:**
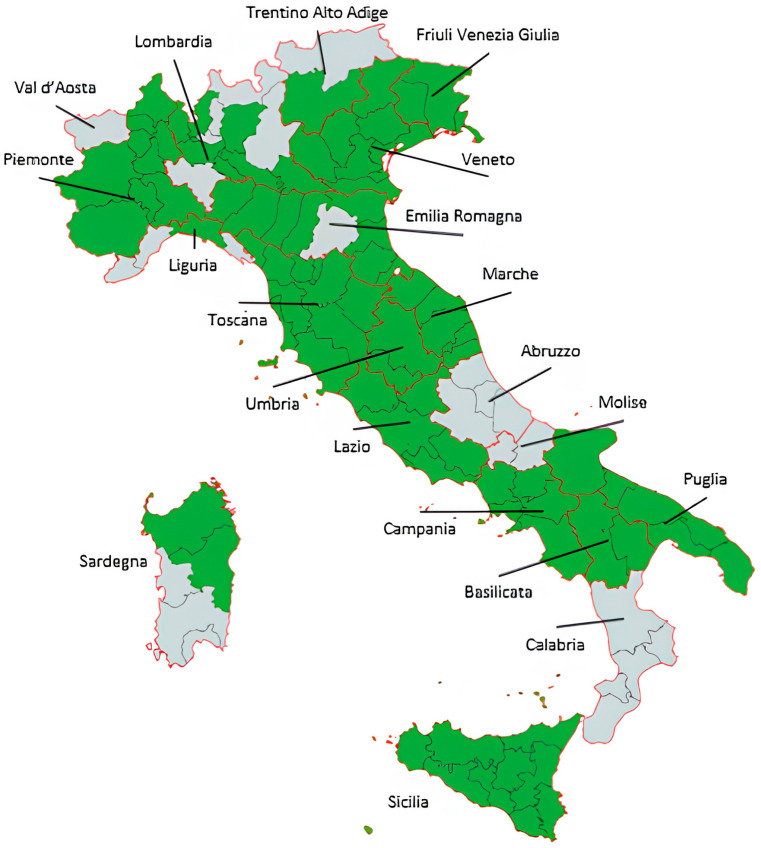
Coverage of the BENCHISTA-Ita project: green areas are covered from the participating PBCRs.

All Italian PBCRs accredited by the AIRTUM were invited to participate in the BENCHISTA-Ita project [20]. A total of 25 Italian PBCRs out of 15 Italian regions (all but Valle d’Aosta, Alto Adige, Abruzzo, Molise, and Calabria) agreed to participate and share at least 3 years of incident cases within the 5-year window, 2013–2017. The participating PBCRs cover about 84% of the Italian child population for the period from 2013 to 2017 (Figure 1).

The participating registries agreed on a depersonalized, patient-level dataset that includes TG stage at diagnosis. The TG includes a two-tiered system to define stage: Tier 1 is the more general stage classification potentially usable by all PBCRs, while Tier 2 is a nested, more detailed staging system intended for use in high-resource settings [4,5]. The full details of the Tier 1 and Tier 2 staging criteria for neuroblastoma are summarized in Table 1 and are available in the Benchista Protocol [3,15]. Variables to be collected by CR are compulsory (demographic variables, ICDO topography and morphology, stage at diagnosis, and examination performed/used for staging, follow-up, and life status information) or optional (additional information regarding NPS, treatment, progression, and cause of death, if any). Standardization in the stage collection among the CR was established by several actions, including the translation into Italian of the TG, online cancer-specific training sessions led by clinical experts, a test on an anonymized set of fictional cases, and the presence of an e-tool available online to all PBCRs [4,5,21].

In this paper, we analyzed data on neuroblastoma diagnosed at 0–14 years of age and during a triennium within the period between 2013 and 2017. Cases were followed up for at least 3 years.

For this specific linkage study, 294 neuroblastoma cases diagnosed between 2013 and 2017 in the population of the participating PBCRs were selected for the linkage.

#### 2.1.2. RINB DB

The Italian Neuroblastoma Registry (RINB) collects data reported by Italian centers recruiting patients with neuroblastoma [18]. RINB uses the diagnostic, staging, and therapy criteria of SIOPEN (International Society of Paediatric Oncology Europe Neuroblastoma Group) [22,23]. RINB follows the rules for stage at diagnosis of the International Neuroblastoma Risk Group Staging System (INRGSS) [24,25,26]. This staging system was also adopted by the TG and was therefore followed by PBCRs.

For this specific pilot study, 578 neuroblastoma cases diagnosed in the period from 2013 to 2017 and collected in the RINB were selected [27].

All NB cases diagnosed or treated in AIEOP centers, which had signed informed consent, were included in the RINB. The RINB database contributed data to the International Neuroblastoma database [28]. The major aim of the RINB is to monitor the efficacy of the clinical network and perform outcome research [29].

### 2.2. Linkage

As a first step, the completeness of selected variables in the BENCHISTA-Ita (called ‘target’ database) and RINB (called ‘source’ database) was assessed, and both DBs were standardized in terms of variable coding. A linkage procedure at the individual level was then applied between the two databases [30,31,32].

We used a probabilistic linkage procedure on R, an open-source software, to make it reproducible and usable in the future by all the registries involved. Due to their completeness and ability to accurately identify patients, the variables in sex, year of birth, province of residence, year of incidence, age in months, hospital of diagnosis, and status of life were considered for the linkage.

**Figure 2:**
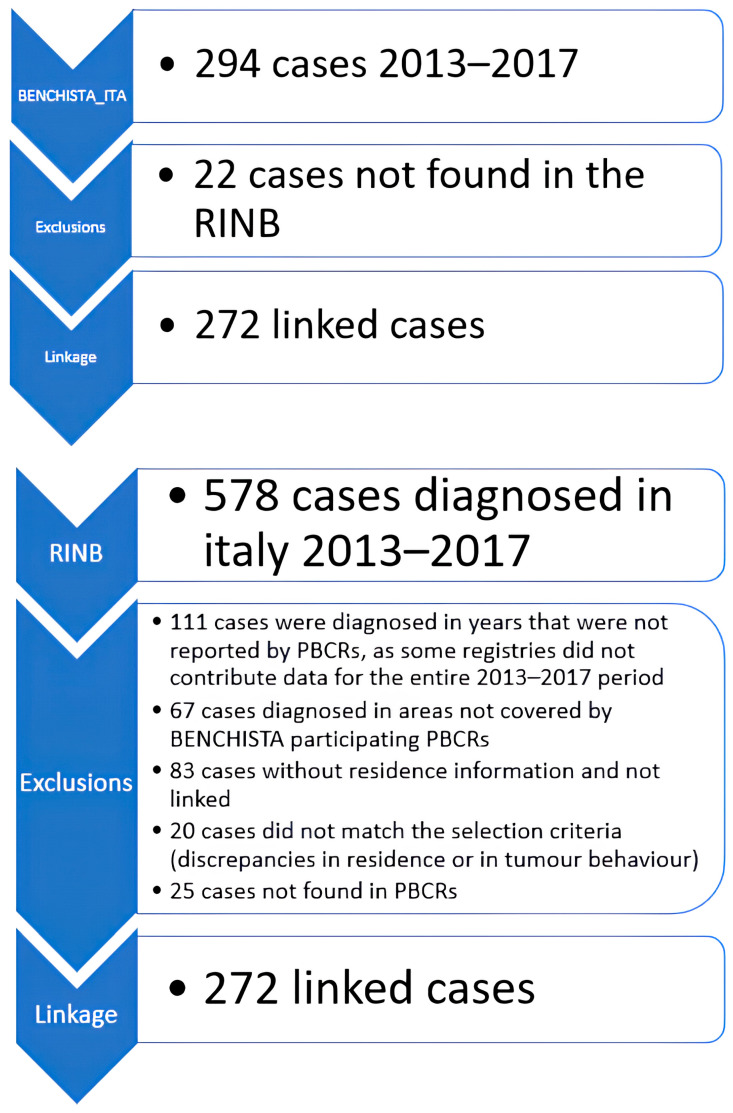
Case selection and exclusion for the linkage in both BENCHISTA-Ita and RINB DBs.

So, we used all 578 RINB cases diagnosed in 2013–2017, including 90 cases (16%, 83 not linked + 7 linked) without the information about patients’ residence. The linkage process is described in Figure 2. A total of 178 cases were excluded because they were diagnosed in years or in areas not covered by the dataset provided by the PBCR. So, 400 cases remained available in RINB and 294 in BENCHISTA-Ita for the linkage. Each record in the target database has been compared with all records in the source database.

This process is divided into two main steps: a deterministic one and a probabilistic one. In the first deterministic step, the link between two records occurs only if the values in each considered variable are the same. Successfully linked records are deemed to refer to the same patient and will not be considered in the next step. In the probabilistic step, a similarity indicator between each target record and all source records is computed. This indicator is expressed as a percentage and calculated by the sum of the similarity of the values found in the two databases for the considered variables. Weights of each variable (values of the weights were 0.15 for year of birth, 0.10 for year of diagnosis, age at diagnosis, sex, status of life, residence, residence code, hospital of diagnosis and province of residence, and 0.05 for the variable region of residence) were empirically assigned thanks to a previous trial setting using Campania and Piedmont PBCR and RINB.

The probabilistic part is divided into several sessions. In each of these sessions, an acceptability threshold is set in such a way that if the similarity function between two records exceeds the acceptability value and the link is unique, the two records are matched. If there was more than one source record that exceeded this threshold, it was placed in a temporary link set, and we manually chose the most correct match based on experience, common sense, and the most likely outcome.

Once identified, for all the matched cases in the two databases, the completeness of the cases in terms of stage, NSP, relapse/recurrence, follow-up and residence information, and concordance for the main characteristics benefiting from the linkage (i.e., stage, NSP, and relapse) were verified. Conflicting information was investigated by contacting our clinical expert and the interested PBCRs [3,16,17].

The version of R used was 2021.09.1+372.

## 3. Results

Of the 294 BENCHISTA-Ita cases, 272 were successfully matched to RINB cases—43 through deterministic linkage and the remaining 229 via probabilistic linkage. Among the probabilistically matched cases, 115 had a matching probability between 0.90 and 0.99, 61 between 0.80 and 0.89, and 53 below 0.80. Of these 53 lower-probability matches, 18 were submitted to the registries for validation. Twenty-two cases were found in the BENCHISTA-Ita and not in the RINB DB. On the other way around, 25 cases were found in the RINB and not in the PBCRs; these 25 cases have been added to the 294 BENCHISTA cases and accounted for in the distributions and completeness calculations (Table 2 and Table 3). In Table 2, the completeness of the variables in the BENCHISTA-Ita and RINB databases and the completeness of the assembled BENCHISTA-Ita DB updated with the clinical information from the RINB DB are reported.

Stage at diagnosis according to TG Tier 2/INRGSS was available in 81% and 98% in BENCHISTA-Ita and RINB, respectively. After the linkage, BENCHISTA-Ita increased its stage completeness from 81% to 99%, from 47% to 86% for N-Myc, and from 68% to 98% for information on relapse or recurrence. Life status and follow-up time since diagnosis were complete for 99% of patients in BENCHISTA-Ita, assuring at least three years of follow-up since diagnosis; by contrast, the follow-up information was present only in 161 out of 272 (59%) of the RINB matched cases. On the other hand, linkage with PBCR data increased the completeness of follow-up from 59% to 99% and of residence, from 97% to 100% in the RINB-matched cases.

Considering RINB as the gold standard for clinical information, such as stage, Table 3, shows the stage distribution of BENCHISTA-Ita data before and after the linkage. Actually, the percentage of missing stage cases decreased from 19% to 1%. The major changes were for locoregional (from 19% to 27%) and metastatic cases (from 29% to 37%).

N-Myc performance information was present in 47% of the BENCHISTA-Ita and updated to 86% when enriching using RINB information.

The information on relapse/progression increased from 68% to 99%.

## 4. Discussion

The major result of linking the BENCHISTA-Ita database with the clinical RINB database, one population-based versus the national clinical database, is the possibility of enriching both databases (Table 2 and Table 3).

First, we improved the degree of completeness of the three main clinical information presented in the BENCHISTA-Ita: stage at diagnosis, relapse/progression, and N-Myc. These data were required by the BENCHISTA-Ita study protocol to help interpret geographic variation in survival. After linkage, the stage at diagnosis was available for all but three cases (1%, see Table 3). However, a small discrepancy in the stage distribution was observed between BENCHISTA-Ita and RINB (Table 3). The difficulty of PBCRs in attributing locoregional (LR) or metastatic stages (M) and thus, in ascertaining the extension and infiltration of the tumor, is due to the failure of PBCRs to access all medical records, including cases from multiple hospital admissions, and to the instrumental test results. A further limitation for PBCRs’ registrars is the difficulty of discussing directly with clinicians and experts, leaving the most complex cases unresolved, which are included in the BENCHISTA-Ita as cases with an “unknown” stage. All these limitations reduce the ability of PBCRs to assign a correct stage at diagnosis according to the TG.

Amplification of the N-Myc gene is one of the major molecular markers in Neuroblastoma, which is associated with a poor prognosis. The limited information on N-Myc in the PBCR database is attributable to the limited availability in clinical documentation of the test requests and, in particular, of the molecular biology reports. In this case, the linkage with the RINB can help PBCRs to complete the clinical information of N-Myc.

Secondly, the comparison between two independent sources, such as BENCHISTA-Ita and RINB, can validate their completeness of incidence and follow-up information, i.e., their ability to include all cases occurring (diagnosed) in a specific period (year) in a defined population (resident in a city, province, or region) and their status of life at a certain time point. These criteria ensure the accuracy of epidemiological indicators and support effective public health planning. Assuming a correct individual match, the linkage procedure actually estimated a collection completeness of 92% (294/319) for the population-based cancer registries and of 93% (297/319) for the clinical RINB. Not all the 25 cases missing in the BENCHISTA-Ita database were actually unknown by the participating PBCRs: ten were codified as tumors without pathological specification, because no clinical documentation was reached by the registries; seven were collected after sending the data to the project, due to delayed receipt of the documentation. So, only eight cases were definitely lost by the PBCR. This validation is particularly valuable in assessing the reliability and robustness of cancer surveillance systems.

We received 578 cases for the period from 2013 to 2017 from RINB, for which we estimated a 93% completeness. Assuming that cases without residence are all Italian, we can estimate an average of 116/0.93 = 124 new cases of NB per year. We estimated that the registration in Italy covers about 85% of the territory. Incidence trend for NB in Italy, as reported by the Italian PBCR in the recent publication [33], remained stable over the recent year.

Thanks to this effort, compared to all the countries included in the BENCHISTA international project [16,17,34], Italy has one of the most complete databases for neuroblastoma, with the percentages of completeness similar to those achieved by registries that link directly with clinical specialized pediatric databases.

This work has also led to the identification of topics in which better standardization of information can be achieved. For example, residence is coded differently between RINB and PBCRs: RINB uses cadastral codes, while PBCRs use ISTAT codes. In our work, this variable had to be transcoded, but in the future, an agreement can be reached to be more efficient in linkage. Moreover, in the RINB, 16% cases do not have information on the residence; therefore, there is room for improvement, even if the restrictions coming from the General Data Protection Regulation (GDPR) might hamper this process, particularly in Italy, where the interpretation of the European Directive in the field of healthcare varies between 20 different regions.

We had to exclude some cases from the linkage because their residential area was not covered by the PBCR (Figure 2). This points to the need for national coverage of pediatric cancer registration in Italy. Some of these patients were diagnosed in regions with active cancer registries (Valle d’Aosta, Alto Adige, and Pavia province) that, however, were not allowed to share the data because of a restrictive interpretation of the GDPR; other were residents in areas (part of the Liguria, Bologna province, Abruzzo, Calabria, and Cagliari province) still needing to start the registration activity. We estimated that the registration in Italy covers about 85% of the territory.

## 5. Conclusions

The BENCHISTA and the BENCHISTA-Ita projects are strengthening collaborative relationships between PBCRs and clinicians not only for the mutual retrieval of information, but also to promote standardized data collection, improve data quality, and support evidence-based decision-making in cancer care. From the clinical point of view, it is crucial to assess if all the children with cancer are accurately treated/included in the clinical protocol. This means to ensure a complete and accurate registration of all the Italian pediatric cases. We recognize that a strict collaboration with the AIEOP can contribute to obtaining a national population-based pediatric cancer registry with the aim of evaluating outcomes, improving the care of children with cancer in the national territory, reducing possible disparities, and providing a surveillance on the incidence trend. A similar situation occurs in Spain, from which an experience using the national pediatric cancer hospital database for a linkage with the Spanish PBCR database was recently published [35]. This experience was developed within the European Joint Action for Rare Cancers [36].

For these reasons, Spain and Italy are well-positioned to establish national registries for childhood cancers, augmented by comprehensive clinical information.

We hope that in the future, the registry will also include data from AIEOP clinical pathology registries—such as the RINB—alongside the current sources, which have mainly been administrative databases, pathology databases, and hospital clinical reports.

We were not able to collect the unique identifier, present and available in Italy, that would allow this linkage to be automatic, due to the general data protection regulations’ interpretation. With the help of patients, advisory groups, and ethical committees, we hope to reduce the impact of these barriers.

## Figures and Tables

**Table 1:** Toronto Guidelines staging criteria for neuroblastoma.

Staging Criteria for Neuroblastoma			
TIER 1		TIER 2	
**Localised**	Localised tumour not involving vital structures and confined to one body compartment	**Stage L1**	Localised tumour that does not involve any viral structures as defined by the list of IDRFs (i.e., there are no IDRFs) and the tumour must be confined within one body compartment, neck, chest, abdomen, or pelvis. An intraspinal tumour extension that does not fulfil the criteria for an IDRF is consistent with stage L1.
**Locoregional**	Locoregional tumour with spread	**Stage L2**	Locoregional tumour with one or more IDRFs. The tumour may be ipsilaterally contiguous within body compartments (i.e., a left sided abdominal tumour with left-sided lung, bone or pleura involvement should be considered stage L2). However, a clearly left sided abdominal tumour with right-sided lung, bone or pleura (or vice versa) involvement is defined as metastatic disease.
**Metastatic**	Distant metastatic disease (except stage MS)	**Stage M**	Distant metastatic disease (i.e., not contiguous with the primary tumour) except as defined for stage MS. Nonregional (distant) lymph node involvement is metastatic disease. However, an upper abdominal tumour with enlarged lower mediastinal nodes or a pelvic tumour with inguinal lymph node involvement is considered locoregional disease. Ascites and/or pleural effusion, even with malignant cells, do not constitute metastatic disease unless they are remote from the body compartment of the primary tumour.
**MS**	Metastatic disease confined to skin, liver, and/or bone marrow in a patient less than 18 months (547 days)	**Stage MS**	Metastatic disease confined to skin, liver, and/or bone marrow in a patient less than 18 months (547 days) MIBG scintigraphy must be negative in bone and bone marrow.

**Table 2:** Completeness of BENCHISTA-Ita and RINB databases.

Variables	BENCHISTA-Ita (294 Cases)	RINB (578 Cases)	RINB (272 Cases)	BENCHISTA-Ita Updated (319 Cases)
TG Tier 1	97%	98%	100%	99%
TG Tier 2	81%	98%	100%	99%
N-Myc	47%	79%	86%	86%
Follow-up	99%	71%	59%	97%
Territorial membership	100%	84%	97%	100%

**Table 3:** Stage distribution of the BENCHISTA-Ita neuroblastoma cases and the BENCHISTA-Ita DB updated using the clinical information available in the RINB.

Variable		BENCHISTA_Ita (294 Cases)	BENCHISTA_Ita Updated (319 Cases)
Stage	localized	23%	24%
	Locoregional	19%	27%
	Metastatic	29%	37%
	Metastatic Special	10%	11%
	unknown	19%	1%
N-Myc	present	21%	24%
	absent	26%	62%
	unknown	53%	14%
Relapse/Recurrence	present	21%	29%
	absent	47%	70%
	unknown	32%	2%

## Data Availability

The datasets presented in this article are not readily available because of privacy issues. Requests to access the datasets should be directed to the participant cancer registries.

## Abbreviations

The following abbreviations are used in this manuscript:

CC	Childhood Cancers
PBCR	Population-Based Cancer Registries
RINB	Italian Neuroblastoma Registry
NSP	Non-Stage Prognostic Factors
AIRTUM	Italian Association of Cancer Registries
IACR	International Agency for Research on Cancer
AIEOP	Italian Association of Pediatric Hematology and Oncology
TG	Toronto Guidelines
DB	Database
INRGSS	International Neuroblastoma Risk Group Staging System
LR	Locoregional
M	Metastatic
AIRC	Italian Association for Cancer Research
